# Patterns of healthcare utilization among patients with sickle cell disease hospitalized with pain crises

**DOI:** 10.1002/jha2.84

**Published:** 2020-10-15

**Authors:** Angie Mae Rodday, Kimberly S Esham, Nicole Savidge, Susan K Parsons

**Affiliations:** 1 Tufts Medical Center, The Institute for Clinical Research and Health Policy Studies, Boston, Massachusetts, USA; 2 Department of Medicine, Tufts University School of Medicine, Boston, Massachusetts, USA; 3 Tufts Medical Center, Hematology and Oncology, Boston, Massachusetts, USA

**Keywords:** clinical research, health services research, sickle cell disease

## Abstract

**Background:**

Vaso-occlusive crises (VOC) are the hallmark of sickle cell disease (SCD). Adults experiencing VOC often have high rates of unexpected healthcare utilization. We characterized prior and future healthcare utilization among adults hospitalized with VOC at an urban, academic medical center.

**Methods:**

We identified 449 VOC hospitalizations among 63 patients from 2013 to 2016. Patients were categorized based on receiving established care at the medical center and prior utilization: (a) not established (n = 21); (b) newly established (n = 10); (c) established with low utilization in past 12 months (<4 VOC hospitalizations) (n = 22); and (d) established with high utilization in past 12 months (≥4 VOC hospitalizations) (n = 10). Patient and hospitalization characteristics and future utilization were compared across categories.

**Results:**

Median age was 26 years (Q1 = 22, Q3 = 29) and 55.6% were female. Established patients with high prior utilization tended to have higher median pain scores at admission (10, *P* = .08). Thirty-day readmissions were highest in established patients with high prior utilization (*P* = .06), but 30-day clinic visits were highest in established patients with low prior utilization (*P* = .08). Adjusted linear regression found that newly established patients (*β* = −4.6, *P* < .01) and established patients with low prior utilization (*β* = −5.6, *P* < .01) had fewer VOC hospitalizations in the ensuing 12 months than established patients with high prior utilization.

**Conclusion:**

Among patients with SCD hospitalized for VOC, there was heterogeneity in healthcare utilization, with persistence in utilization over time for some patients. Efforts are needed to shift care from the acute setting to the outpatient clinic, which may lead to improved outcomes.

## 1 | INTRODUCTION

Sickle cell disease (SCD) is the most common inherited blood disorder in the United States, but primarily affects people of African descent and Hispanics of Caribbean ancestry [1]. Patients with SCD have one of the highest 30-day hospital readmission rates (>30%) of all chronic conditions, reflecting both the severity of their condition and potentially avoidable healthcare utilization [2–4]. Vaso-occlusive crisis (VOC), also referred to as “painful crisis,” is the hallmark of SCD and the most common reason for hospitalization among patients with SCD [5,6]. In addition to VOC, patients with SCD also experience hemolytic anemia and organ system complications, which all contribute to morbidity, poor health-related quality of life, high healthcare utilization, and premature mortality. Although a widely used and validated measure of SCD severity does not exist, frequent VOCs and more SCD-related complications (ie, acute chest syndrome, pneumonia, avascular necrosis of bone) likely represent higher disease severity [7,8].

Current treatments for VOC are primarily palliative and may involve pain management with or without opioids, while treatment with hydroxyurea or blood transfusions may reduce VOC occurrence. More recently, the United States of America (USA) Food and Drug Administration (FDA) has approved additional drugs (eg, l-glutamine, crizanlizumab, voxelotor) to prevent VOC; however, their uptake into clinical practice is not well understood [9]. Most patients with SCD manage their pain at home and have infrequent emergency department (ED) and hospital visits [10]. However, healthcare utilization is skewed with the highest utilization and healthcare costs among patients with severe disease, such as those with more SCD-related complications [10]. When patients in VOC present acutely to EDs or clinicians’ offices, they have usually exhausted home care options, which signals the need for more aggressive pain management, such as parenteral opioids delivered in the inpatient setting [11]. Unfortunately, patients with SCD face barriers to appropriate pain management due to clinician mistrust, stigmatization, or fear of drug use [12–17].

A subset of patients with SCD may experience persistent healthcare utilization over time, while others may have intermittent utilization reflecting changing disease or patient factors [18]. Types of healthcare utilization may also vary across patients or over time. For example, a patient who has routine clinic visits with their hematologist and a well-delineated home-based care plan may have better disease management and avoid ED visits or unexpected, prolonged hospital stays. However, evidence suggests that patients with SCD have worse health outcomes and access to fewer health resources compared to other diseases [19–22]. In addition, patients in racial/ethnic minority groups have worse access to effective primary care and are more likely to receive low-value services [23,24]. Previous research has shown that not receiving follow-up care, missing appointments, and not having a primary care physician were associated with more 30- day readmissions for patients with SCD [25–27]. Other factors associated with higher healthcare utilization in adults with SCD were SCD complications, mood disorders, fragmented care, and VOCs [18,25,26].

Our institution (Tufts Medical Center in the city of Boston, Massachusetts (MA), USA) identified patients with SCD as one of the patient populations with the highest 30-day readmissions rate, indicating an opportunity for quality improvement among a vulnerable group of patients who have historically faced disparities in their care. Identifying the subset of patients with SCD who are at risk for high healthcare utilization could enhance targeting of services and improve disease management. Therefore, this study aimed to create a cohort of patients with SCD hospitalized for VOC to describe the persistence of different types of healthcare utilization over time and identify factors associated with healthcare utilization. We hypothesized that patients with more severe disease and high prior utilization would have more healthcare utilization in the future.

## 2 | METHODS

### 2.1 | Sample

**FIGURE 1:**
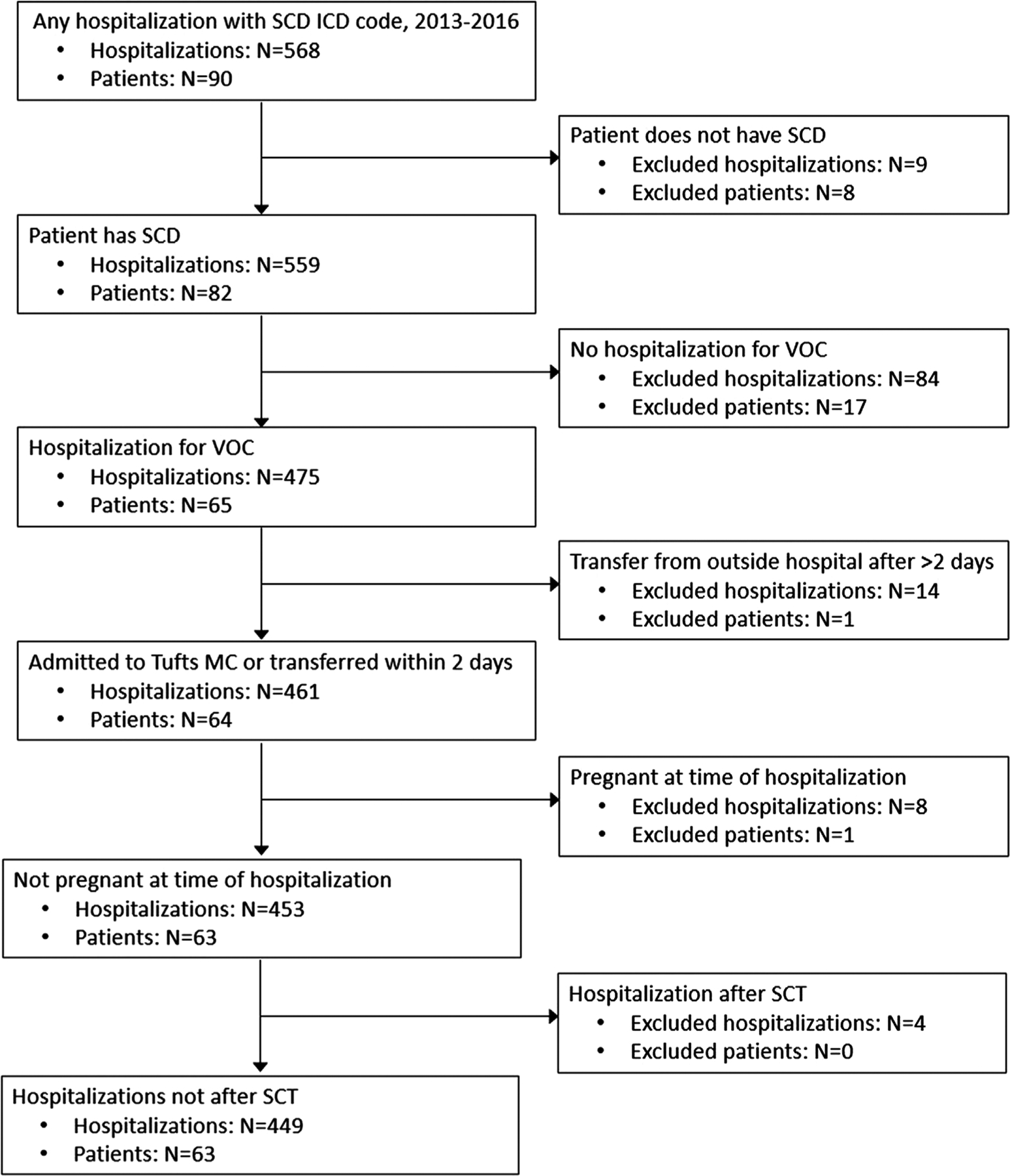
Cohort development at hospitalization and patient level

We created a retrospective cohort of consecutive hospitalizations for VOC among adult patients (≥18 years) with SCD from 2013 to 2016 at Tufts Medical Center, an academic medical center in the city of Boston, MA, USA. Hospital billing data were used to identify patients and hospitalizations with any ICD-9 or ICD-10 codes for SCD (ie, 282.6 or D57). Using this list of potentially eligible patients and hospitalizations generated from hospital billing data, trained study staff reviewed the electronic medical record (EMR) to determine eligibility for each patient and hospitalization. Patients had to have a confirmed diagnosis of SCD; all SCD genotypes (hemoglobin (Hb) SS, Hb SC, Hb S*β*+ thalassemia, Hb S*β*0 thalassemia, and other/unspecified), with the exception of sickle-cell trait, were included. Only hospitalizations for VOC were included; that is, hospitalizations for procedures (ie, cholecystectomy) were excluded, as were all hospitalizations during pregnancy where the pain experience and treatment may differ. We also excluded transfer hospitalizations if the patient spent > 2 days at an outside hospital, as this would limit available information about the beginning of the pain crisis. Any hospitalizations occurring after a patient’s stem cell transplant (SCT) were also excluded because SCT is the one available cure for SCD. Given that patients could have multiple hospitalizations, it is possible that some of a patient’s hospitalizations were excluded, while others were included; patients with no eligible hospitalizations were excluded (Figure 1). This study was approved by the Tufts Health Sciences Institutional Review Board.

### 2.2 | Data

The study database was designed to have three levels of information: patient, hospitalization, and hospital day. Hospital days were nested in hospitalizations, which were subsequently nested within patient. Hospital billing data provided hospitalization-level information on admission date, discharge date, discharge status, insurance, visit type (eg, inpatient, outpatient, ED), and hospital service (pediatric, adult). Insurance was categorized as any Medicare, Medicaid (without Medicare), or private. In the USA, Medicare is the national health insurance program for Americans aged ≥65 years and a small subset aged <65 years who qualify based on certain medical conditions or disability status. Medicaid is a federal and state program that helps cover medical costs for patients with limited income and resources. Private insurance is typically offered through employers, although individual coverage can also be acquired through marketplaces [28]. Length of stay was calculated as discharge date minus admission date plus 1. Trained study staff abstracted additional patient, hospitalization, and hospital day information from the EMR.

Patient-level data included date of birth, gender, race/ethnicity, socio-economic status (SES), and SCD genotype (categorized as Hb SS, Hb SC, other), which were all abstracted from the EMR. As a proxy for SES, we calculated the Area Deprivation Index (ADI) for each patient based on their street address, which is easily available from the EMR [29]. The ADI is a well-established method for quantifying socio-economic disadvantage that combines 17 USA Census block indicators of poverty, education, housing, and employment [30,31]. ADI scores have been associated with hospital readmission and mortality in the general population and in chronic diseases, but have not been applied to SCD specifically [32]. Patients’ street addresses were used to identify the associated nine-digit zip codes that generated ADI scores, which were assigned state-level ADI deciles for Massachusetts. State-level census block ADI scores are ranked from lowest to highest and assigned deciles of 1 to 10, where higher scores indicate more disadvantage.

Hospitalization-level data abstracted from the EMR included whether the patient was established at Tufts Medical Center, history of SCD complications, documented affective disorder in the prior 12 months (for established patients only), treatments received during the hospitalization, and home medications, including hydroxyurea and home-based pain regimens. At each hospitalization, patients were defined as established at Tufts Medical Center if they either had a hematology clinic visit in the prior 12 months, or there was documentation in the EMR that the patient was established. Newly established patients were defined as those without a prior hematology clinic visit in the 12 months before that hospitalization, but at least one documented hematology clinic visit in the 12 months following that hospitalization. A list of 13 SCD complications (stroke; osteonecrosis/avascular necrosis; cholecystectomy or cholecystitis; hyper-hemolysis/hyper hemolytic syndrome; acute renal failure or chronic kidney disease; pulmonary hypertension or acute chest syndrome; priapism; retinopathy; silent infarct lesion on MRI; sickle hepatopathy; splenectomy, hypersplenism, splenic sequestration; thrombosis; leg or foot ulcer/sores) was developed, based on known SCD complications and those included in the Adult.

Sickle Cell Quality of Life Measurement Information System (ASCQ-Me) SCD Medical History Checklist [7]. SCD complications that were documented in the EMR in the 12 months prior to hospitalization were included as a marker of disease severity; each complication type was only recorded once (eg, yes/no acute chest syndrome), so the number of SCD complications reflects the different types of complications experienced by the patient, not the number of episodes of a given complication. Two hematologists reviewed each patient’s SCD complications after extraction by study staff. Medications were categorized as opioids, non-opioid pain medications, disease modifying medications (eg, hydroxyurea), and transfusion support. Hospital day-level data included use of opioids and all available pain scores rated on a numeric rating scale (0 = no pain; 10 = worse pain), which was abstracted from the EMR.

In addition to VOC hospitalizations, healthcare utilization of hematology clinic visits and ED visits was generated from hospital billing data, then verified by EMR review. Healthcare utilization data were extracted for all eligible patients during the study period (2013–2016) with a 12-month look back period to 2012 to capture prior healthcare utilization. VOC hospitalizations from outside hospitals from 2012 to 2016 that were documented in our EMR were included when calculating the number of prior or future VOC hospitalizations.

### 2.3 | Analysis

Patients’ entry into our cohort, defined by their first hospitalization at Tufts Medical Center in the study window, is the focus on the current analyses. At their first hospitalization, patients were categorized as (a) not established, (b) newly established, (c) established with low utilization in the prior 12 months (<4 VOC hospitalizations), and (d) established with high utilization in the prior 12 months (≥4 VOC hospitalizations). The cut-off of 4 was based on prior research indicating the clinical importance of four VOC hospitalizations per year; [33] this cut-off is slightly higher than some cut-offs of the number of VOCs or VOC-related healthcare utilization because our sample was more severe with at least one hospitalization in the study period [34].

We calculated the following types of 30-day utilization after discharge from the first hospitalization using hospital billing data: 30-day unplanned readmission rate, 30-day ED visit rate, 30-day hematology clinic visit rate, and any 30-day utilization (hospitalization, ED visit, or clinic visit). Healthcare utilization was also calculated for the 12 months following the first hospitalization using hospital billing data, including number of VOC hospitalizations, cumulative VOC hospitalization days (calculated by adding all hospitalized days), number of ED visits, number of hematology clinic visits, and total healthcare contact days (calculated by adding VOC hospitalization days, ED visits, and hematology clinic visits; multiple visits on 1 day were only counted once).

Data were described using summary statistics, including means, standard deviations (SD), medians, 25th and 75th quartiles (Q1, Q3), frequencies, and percentages. Patient characteristics (eg, demographic and clinical factors) and hospitalization characteristics (eg, length of stay, admission and discharge pain scores, opioid treatment) were described across the four categories of patients defined by established care and prior utilization. Future healthcare utilization and persistence of healthcare utilization over time was described for established and newly established patients only, as patients who were never established would not be expected to seek future healthcare at our institution. Statistical comparisons were made across the groups using the Fisher exact test (categorical variables) or Kruskal-Wallis test; nonparametric tests were used to account for the small sample sizes and non-normal distribution of some variables. A linear regression model was fit to assess the association between future total healthcare contact days and prior utilization, restricted to established and newly established patients. Our sample size limited how many variables could be included in the linear regression model, so we adjusted for hydroxyurea use and SCD complications, which we hypothesized would be the most important confounders. Model assumptions were assessed with residual plots. Data were analyzed using SAS Enterprise Guide 7.1 (SAS Institute, Inc. Cary, NC) and used a two-sided alpha of 0.05.

## 3 | RESULTS

### 3.1 | Cohort development

Initially, 568 hospitalizations among 90 patients with SCD were identified as potentially eligible from 2013 to 2016. The final cohort included 449 hospitalizations at Tufts Medical Center for VOC among 63 patients (Figure 1). Reasons for exclusion were: patient did not have SCD (9 hospitalizations excluded, 8 patients excluded), no VOC (84 hospitalizations excluded, 17 patients excluded), transfer from outside hospital after 2 days (14 hospitalizations excluded, 1 patient excluded), pregnancy (8 hospitalizations excluded, 1 patient excluded), and hospitalization after SCT (4 hospitalizations excluded, 0 patients excluded). The median number of hospitalizations per patient over the full study period was 4 (Q1 = 1, Q3 = 10).

### 3.2 | Patient characteristics

Overall median age was 26 years (Q1 = 22, Q3 = 29) with a maximum age of 57 years; 55.6% were female (Table 1). Most patients were cared for on the adult hematology service (84.1%). At their first hospitalization in the study window, 21 patients were categorized as not established, 10 as newly established, 22 as established with low prior utilization, and 10 as established with high prior utilization. Across these four categories, the established with high prior utilization group was more likely to be female (80.0%), have Medicaid without Medicare insurance (80.0%), have Hb SS genotype (80.0%), and be prescribed hydroxyurea (80.0%). Of these variables, only female reached statistical significance (*P* = .04). Among established patients, those with high prior utilization were more likely to have an affective disorder (80.0%) than those with low prior utilization (45.5%), but this did not reach statistical significance (*P* = .12)

### 3.3 | Hospitalization characteristics

The median length of hospital stay was 7 days (Q1 = 4, Q3 = 10) for the first hospitalization in the study window (Table 2). Across the four categories of patients defined by established care and prior utilization, those who were established with high prior utilization had the highest median pain score at admission (10, Q1 = 9, Q3 = 10, *P* = .08) and discharge (5, Q1 = 4, Q3 = 6, *P* = .25), but neither reached statistical significance. During the hospitalization, the majority of patients (85.5%) were treated with opioids.

### 3.4 | Future utilization

Analyses of future utilization were restricted to established and newly established patients (n = 42) (Table 3). In the 30 days following hospital discharge, 69.1% of patients had some type of utilization, with no statistically significant differences across groups. Thirty-day readmissions were more common among established patients with high prior utilization (60.0%) and the newly established patients (40.0%), compared to established patients with low prior utilization (18.2%) (*P* = .06). On the other hand, established patients with low prior utilization tended to have more clinic visits within 30 days (63.6%) compared to established patients with high prior utilization (40.0%) and newly established patients (20.0%) (*P* = .08).

**FIGURE 2:**
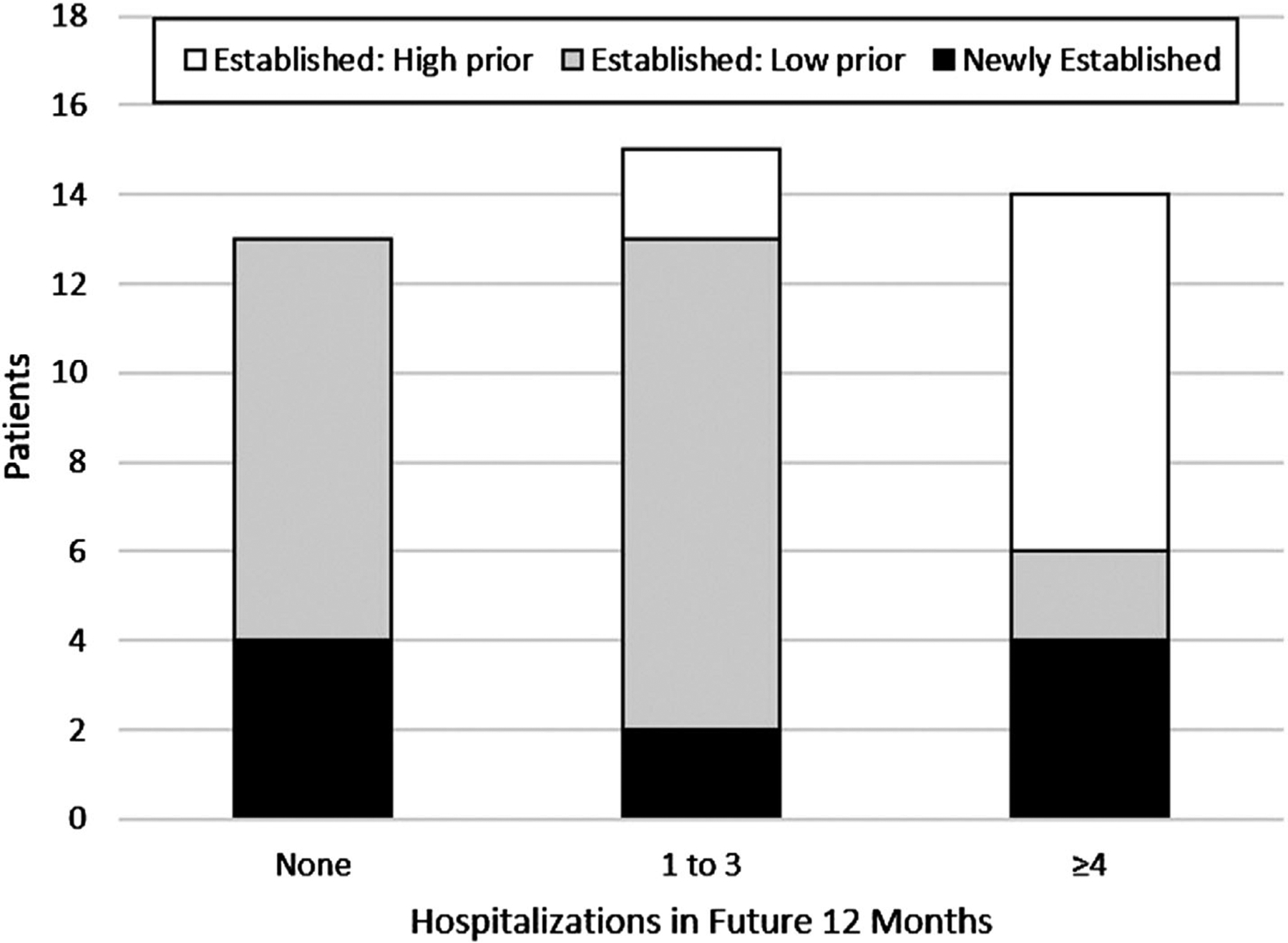
Persistence of VOC-related hospitalizations over time by prior utilization among established and newly established patients, n = 42

In the 12 months following hospital discharge, established patients with high prior utilization continued to have high utilization (Table 3, Figure 2). For example, established patients with high prior utilization had a median of 78 future total healthcare contact days (Q1 = 58, Q3 = 110), compared to 10 days (Q1 = 5, Q3 = 17) in established patients with low prior utilization, and 8.5 days (Q1 = 3, Q3 = 35) in newly established patients (*P* < .01). Similarly, established patients with high prior utilization had a median of 7 future VOC-related hospitalizations (Q1 = 4, Q3 = 8), compared to 1 hospitalization (Q1 = 0, Q3 = 2) in established patients with low prior utilization, and 1.5 hospitalizations (Q1 = 0, Q3 = 3) in newly established patients (*P* < .01). Similar results were found in the adjusted linear regression model where established patients with low prior utilization (*β* = −5.6, SE = 1.1, *P* < .01) and newly established patients (*β* = −4.6, SE = 1.4, *P* < .01) had fewer future VOC-related hospitalizations than established patients with high prior utilization (Table 4). Although hydroxyurea use (*β* = 2.1, SE = 1.2, *P* = .09) and SCD complications (*β* = 0.8, SE = 0.4, *P* = .08) approached statistical significance in unadjusted results, they were not significant in the adjusted model (*P* = .43 and *P* = .21, respectively).

## 4 | DISCUSSION

We successfully created a cohort of 449 hospitalizations for VOC among 63 patients with SCD at a single urban academic medical center from 2013 to 2016. Even among this high-severity group of patients, there was still heterogeneity in healthcare utilization, with persistence in utilization over time for some patients. Established patients with high prior utilization had more 30-day readmissions, but had fewer 30-day clinic visits compared to established patients with low prior utilization, which may reflect an opportunity to improve care delivery.

For most patients with SCD, healthcare utilization and hospitalizations rates are low [34]. In our sample of hospitalized patients with SCD, only a subset had persistent high utilization over time. The availability heuristic may explain the inappropriate use of terms like “frequent flyer” because providers may more easily recall this subset of patients, despite their lack of representativeness of all patients with SCD [15,35]. Pain severity and frequency may be underestimated by clinicians in patients without high utilization, despite many patients still experiencing pain outside of the healthcare setting [36]. Although prior research shows that patients with more SCD complications typically constitute the subset with higher utilization [10,18], we found a relatively comparable number of complications across prior utilization groups. Given that our sample all experienced at least one VOC hospitalization, they may represent a generally more severe group, or there may be other factors influencing healthcare utilization [37].

As with other chronic health conditions, the management of SCD may be further complicated by the patient’s mental health status. Over 50% of established patients in our sample had a documented affective disorder, which is comparable to rates reported in other SCD samples [38,39]. Affective disorders and the pain experience are deeply intertwined, with more pain reported among those with anxiety and depression, and pain leading to symptoms of anxiety and depression [39]. Patients with high prior utilization had nearly twice the rate of affective disorders than patients with low prior utilization; affective disorders were not reported for not established and newly established patients given the lack of documented medical history at the time of their first hospitalization. Prior research has similarly found that patients with SCD and mental health disorders have higher healthcare utilization than patients without mental health disorders [37,38].

Factors related to SES, insurance, and employment may also be related to healthcare utilization among patients with SCD [40]. As VOC episodes disrupt daily functioning, obtaining or maintaining employment may be difficult. The inability to work may lead to limited income, lack of employer-based private insurance, housing instability, barriers to affording medical care and medications, and difficulties caring for themselves and their family. In fact, in our non-elderly cohort with a median age of 26 years, >25% had Medicare insurance, indicating that they qualified based on disability status and were unable to work due to their disease. Medicaid, which is available to patients with limited income and resources, was the most common insurance in established patients with high prior utilization. Although Medicaid can reduce patient cost-sharing, patients with Medicaid have less access to primary care than privately insured patients, which could be a marker of fragmented care [41]. Investing resources for patients to navigate the disability process for Medicare may improve access to affordable healthcare [42]. As a proxy measure for SES, we found that the ADI did not vary by prior utilization level. However, the median overall ADI score of 6.5 was higher than the statewide median of 5, providing further evidence of the SES challenges faced by many patients with SCD. Other measures of SES, such as employment, housing, or income, were not routinely collected in the EMR, so could not be studied, despite their known relationship with healthcare utilization [40].

Research has suggested several approaches for reducing avoidable healthcare utilization in patients with SCD, such as the use of individual pain treatment plans when patients present to the ED with a VOC, and shorter time to opiate dosing [26,43]. Shifting care from the reactionary acute setting to proactive disease management provided in the clinic may also reduce avoidable utilization. Our analysis found similar rates of any type of 30-day healthcare utilization across established patients with low and high prior utilization, but a higher 30-day readmission rate in the high prior utilization group and a higher 30-day clinic visit rate in the low prior utilization patients. In addition, use of hydroxyurea or chronic transfusions may result in fewer VOC episodes, shorter VOC hospitalizations, fewer SCD-related hospitalizations, and lower opioid utilization [44–47]. However, we found the highest rate of hydroxyurea prescription in the established high prior utilization group. One explanation is patients with higher utilization may have more severe disease and thus, be more likely to have already been identified as potential candidates for treatment with hydroxyurea. In addition, having received a prescription for hydroxyurea does not mean patients were adherent, which we were unable to assess. Further, patients of lower SES may experience cost-related medication non-adherence [48]. Prior research has shown wide variability in rates of hydroxyurea adherence, and those with higher healthcare utilization perceive hydroxyurea as less useful, which may lower adherence [49,50]. With regards to chronic transfusion, we had an insufficient number of patients receiving chronic transfusions to analyze its association with future healthcare utilization. Although chronic transfusion are indicated for stroke prevention in pediatric patients with SCD, standards for chronic transfusion protocols among adult patients with SCD are less established; benefits and risks, including the burden of chronic transfusions, should be considered [46,51]. Although not available during the current study, the FDA has recently approved additional drugs (eg, l-glutamine, crizanlizumab, voxelotor) to prevent VOC [9]. Their increased uptake into clinical practice could potentially reduce suffering associated with VOC as well as preventable healthcare utilization.

A small subset of patients hospitalized in our cohort became newly established at Tufts Medical Center around the time of hospitalization and another one-third of patients were not established and did not become established. Newly established patients were a heterogeneous group in terms of patient and hospitalization characteristics and future utilization. A prior study of SCD hospitalizations and ED visits found that adults and patients with public or no insurance were more likely to receive care at multiple hospitals, reflecting fragmented care [52]. Patients with SCD have experienced barriers to receipt of appropriate pain management due to clinician mistrust, stigmatization, and fear of drug abuse, which may further contribute to fragmented care and use of different healthcare providers [12–17]. Like other urban areas with multiple acute care hospitals, patients in our region (Boston, MA) had many options when seeking acute care, particularly if one hospital was geographically closer in the event of a pain crisis. Hospitalizations with non-established patients who lack routine SCD care present an opportunity to connect patients with care. In addition, despite only including adults over age 18, we identified a subset of patients who were cared for on the pediatric hematology service. This reflects challenges and reluctance of some patients to fully transition to adult care [53].

We acknowledge this study’s limitations. Although the sample size was small and some analyses may have been under powered, we were able to identify important factors that may influence high healthcare utilization and should be the focus of future, larger studies. Our sample size was further reduced by the exclusion of patients not established at our medical center from some analyses. This further highlights issues related to fragmented care for SCD. This study was conducted at a single medical center, which may reduce generalizability. It is possible that some utilization was missed and our results are underestimates of actual utilization. Patients presenting at other institutions may have both low prior and future hospitalizations; however, our inclusion of VOC hospitalizations at outside hospitals that were documented in the EMR should help reduce this bias. We did not collect information on laboratory markers, hematocrit or ferritin level, which could be associated with increased VOC and future healthcare utilization [8]. However, changes in these laboratory markers can be patient dependent and may require future research.

In conclusion, we created a cohort of patients with SCD hospitalized for VOC and described their healthcare utilization prior to and following hospitalization for an acute VOC episode. A subset of patients had high healthcare utilization that persisted over time. Differences in types of future utilization (eg, readmissions, clinic visits) represent an opportunity to shift care from the acute setting to the outpatient clinic setting. Issues related to mental health, access and adherence to hydroxyurea, and fragmented care may also contribute to avoidable, acute healthcare utilization. Although addressing these issues will be challenging, particularly given patient-reported mistrust and barriers to pain management, it should help improve disease management and result in better clinical outcomes.

## Figures and Tables

**TABLE 1:** Patient characteristics at time of first hospitalization by established care and prior utilization, n = 63

	Overall, n = 63	Never established, n = 21	Newly established, n = 10	Established: low prior utilization, n = 22	Established: high prior utilization, n = 10	*P*-value
Age, median (Q1, Q3)	26 (22,29)	26 (23,31)	24.5 (23, 27)	23.5 (20, 33)	25.5(19, 32)	.60
Female, n (%)	35 (55.6%)	7 (33.3%)	5 (50.0%)	15 (68.2%)	8 (80.0%)	.04
Race/ethnicity, n (%)						.25
Black/African American	49 (77.8%)	18 (85.7%)	8 (80.0%)	17 (77.3%)	6 (60.0%)	
Hispanic	12 (19.1%)	2 (9.5%)	1 (10.0%)	5 (22.7%)	4 (40.0%)	
Other	2 (3.2%)	1 (4.8%)	1 (10.0%)	0 (0.0%)	0 (0.0%)	
Insurance, n (%)						.84
Any Medicare	18 (28.6%)	6 (28.6%)	3 (30.0%)	7 (31.8%)	2 (20.0%)	
Medicaid/No Medicare	38 (60.3%)	13 (61.9%)	5 (50.0%)	12 (54.6%)	8 (80.0%)	
Private only	7 (11.1%)	2 (9.5%)	2 (20.0%)	3 (13.6%)	0 (0.0%)	
SCD genotype, n (%)						.28
Hb SS	39 (61.9%)	11 (52.4%)	5 (50.0%)	15 (68.2%)	8 (80.0%)	
Hb SC	13 (20.6%)	3 (14.3%)	3 (30.0%)	5 (22.7%)	2 (20.0%)	
Other^a^	11 (17.5%)	7 (33.3%)	2 (20.0%)	2 (9.1%)	0 (0.0%)	
SCD Complications, median (Q1, Q3)	3 (2, 3)	3 (1, 3)	2.5 (1,4)	3 (2, 3)	3 (2,4)	.79
Affective disorder, n (%)^b^	23 (36.5%)	n/a	n/a	10 (45.5%)	8 (80.0%)	.12
Prescribed hydroxyurea, n (%)^c^	40 (64.5%)	13 (61.9%)	4 (44.4%)	15 (68.2%)	8 (80.0%)	.44
ADI score, median (Q1,Q3)^d^	6.5 (6, 8)	6 (5,8)	6 (6, 9)	7 (6,8)	7 (6, 7)	.93

Abbreviations: ADI, Area Deprivation Index; Hb, hemoglobin; SCD, sickle cell disease.

a Other includes Hb S*β*+ thalassemia, Hb S*β*0 thalassemia, other.

b Affective disorders only defined for established patients, *P*-value from comparison of these two groups.

c One newly established patients did not have information in the EMR about their home medication regimen.

d Seven patients were missing ADI score.

**TABLE 2:** Characteristics of first hospitalization by established care and prior utilization, n = 63

	Overall, n = 63	Never established, n = 21	Newly Established, n = 10	Established: low prior utilization, n = 22	Established: high prior utilization, n = 10	*P*-value
Length of stay in days, median (Q1, Q3)	7 (4,10)	5 (3, 9)	8.5 (4,12)	6.5 (5, 9)	9 (8,15)	.21
Admission pain score, median (Q1, Q3)	9 (8,10)	9 (8,10)	9 (8,10)	8 (7, 9)	10 (9,10)	.08
Discharge pain score, median (Q1, Q3)	4 (2, 6)	4 (2, 6)	4 (3, 6)	2.5 (1, 5)	5 (4, 6)	.25
Treated with opioid, n (%)	53 (85.5%)	20 (95.2%)	6 (66.7%)	18 (81.8%)	9 (90.0%)	.18

**TABLE 3:** Future healthcare utilization following first hospitalization by prior utilization among established and newly established patients, n = 42

	Overall, n = 42	Newly Established, n = 10	Established: low prior utilization, n = 22	Established: high prior utilization, n = 10	*P*-value
30-Day Utilization					
30-day readmissions, n (%)	14 (33.3%)	4 (40.0%)	4 (18.2%)	6 (60.0%)	.06
30-day ED visit, n (%)	7 (16.7%)	3 (30.0%)	2 (9.1%)	2 (20.0%)	.41
30-day clinic visit, n (%)	20 (47.6%)	2 (20.0%)	14 (63.6%)	4 (40.0%)	.08
30-day any utilization, n (%)	29 (69.1%)	6 (60.0%)	15 (68.2%)	8 (80.0%)	.62
12-Month Future Utilization					
VOC-related hospitalizations, median (Q1, Q3)	2 (0,4)	1.5 (0, 3)	1 (0,2)	7 (4,8)	<.01
Cumulative VOC-related hospital days, median (Q1, Q3)	7.5 (0,37)	3.5 (0, 29)	5.5 (0,12)	57 (46,84)	<.01
ED visits, median (Q1, Q3)	2 (1,6)	2 (1, 7)	1 (1,3)	6.5 (4,8)	<.01
Hematology clinic visits, median (Q1, Q3)	5 (2,10)	2.5 (1,4)	5 (2,10)	12.5 (10,19)	<.01
Total healthcare contact days,^a^ median (Q1, Q3)	16 (5, 36)	8.5 (3, 35)	10 (5,17)	78 (58,110)	<.01

Abbreviations: ED, emergency department; VOC, vaso-occlusive crisis.

a Total healthcare contact days calculated by adding VOC hospitalization days, ED visits, and hematology clinic visits; overlap days were removed.

**TABLE 4:** Linear regression model for future VOC-related hospitalizations among established and newly established patients, n = 42

	Unadjusted		Adjusted	
	*β* (SE)	*P*-value	*β* (SE)	*P*-value
Prior utilization/established status				
Newly Established	−5.1 (1.3)	<.01	−4.6 (1.4)	<.01
Established: low prior utilization	−5.8 (1.1)	<.01	−5.6 (1.1)	<.01
Established: high prior utilization	Reference		Reference	
Hydroxyurea use	2.1 (1.2)	.09	0.9 (1.1)	.43
SCD complications	0.8 (0.4)	.08	0.5 (0.4)	.21

Abbreviations: SCD, sickle cell disease; VOC, vaso-occlusive crisis.

## Abbreviations:

SCD: sickle cell disease
VOC: vaso-occlusive crisis
